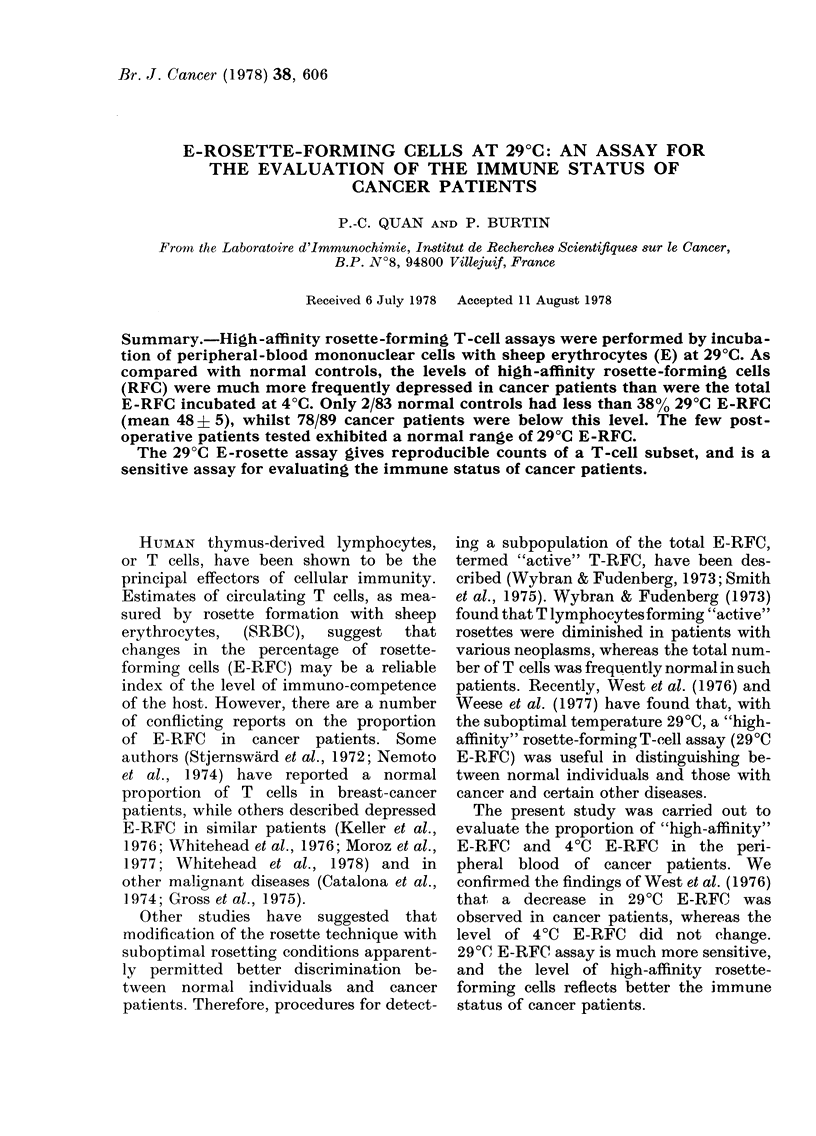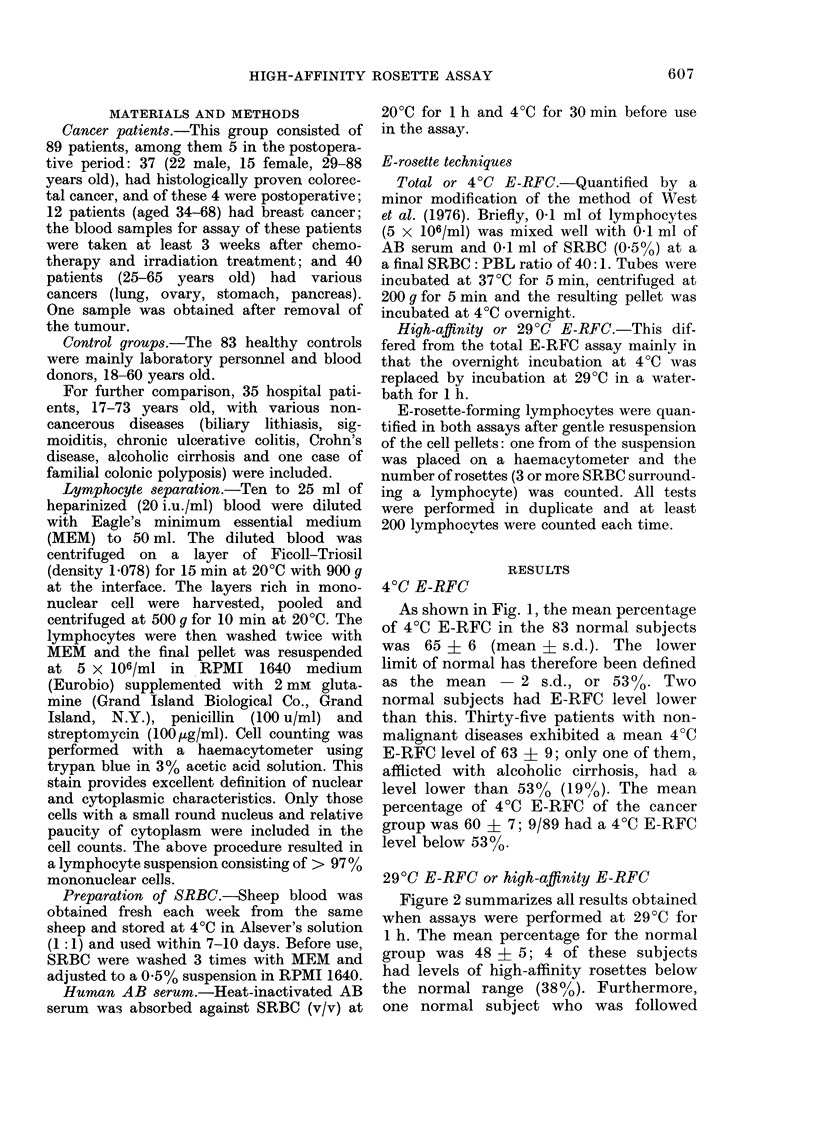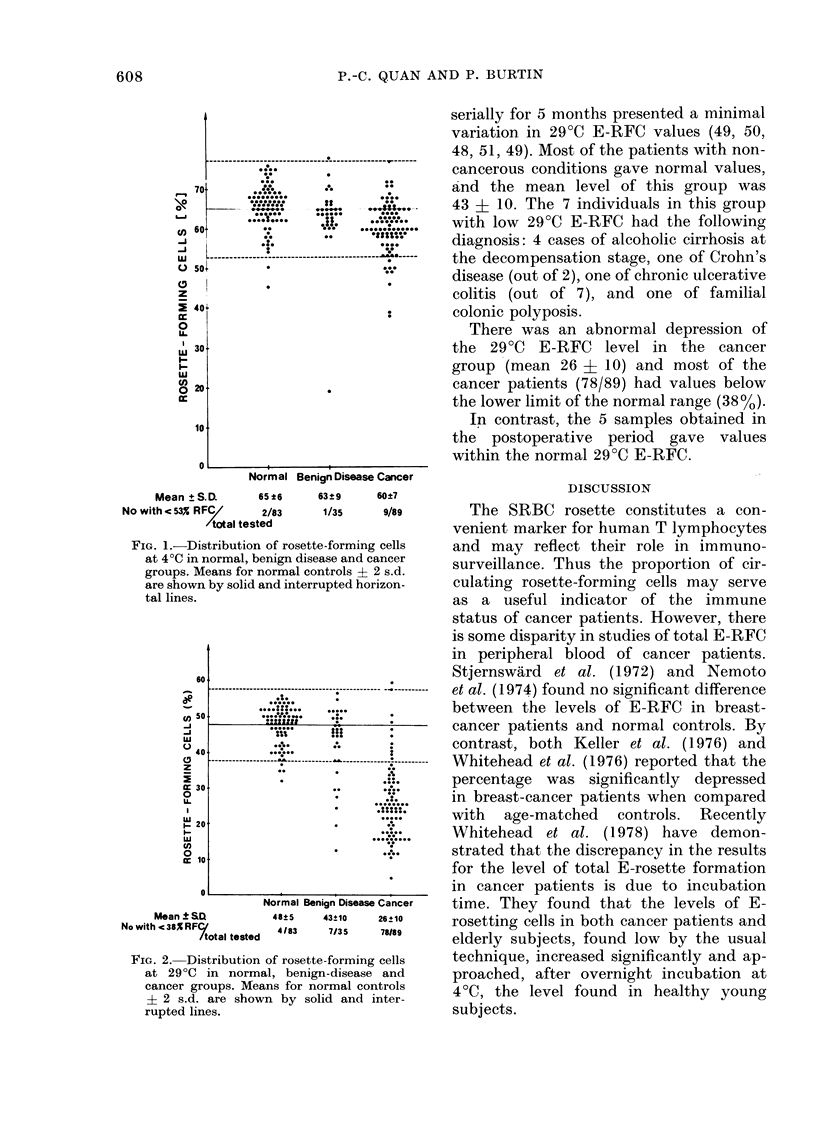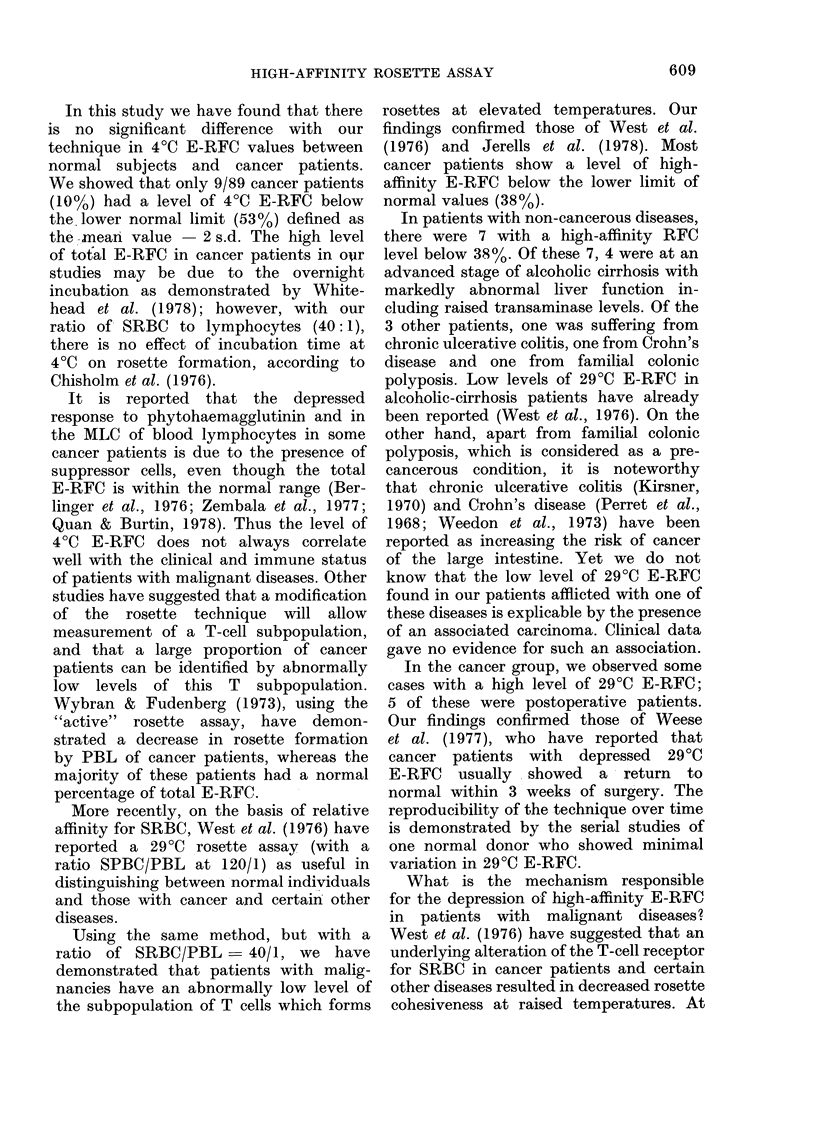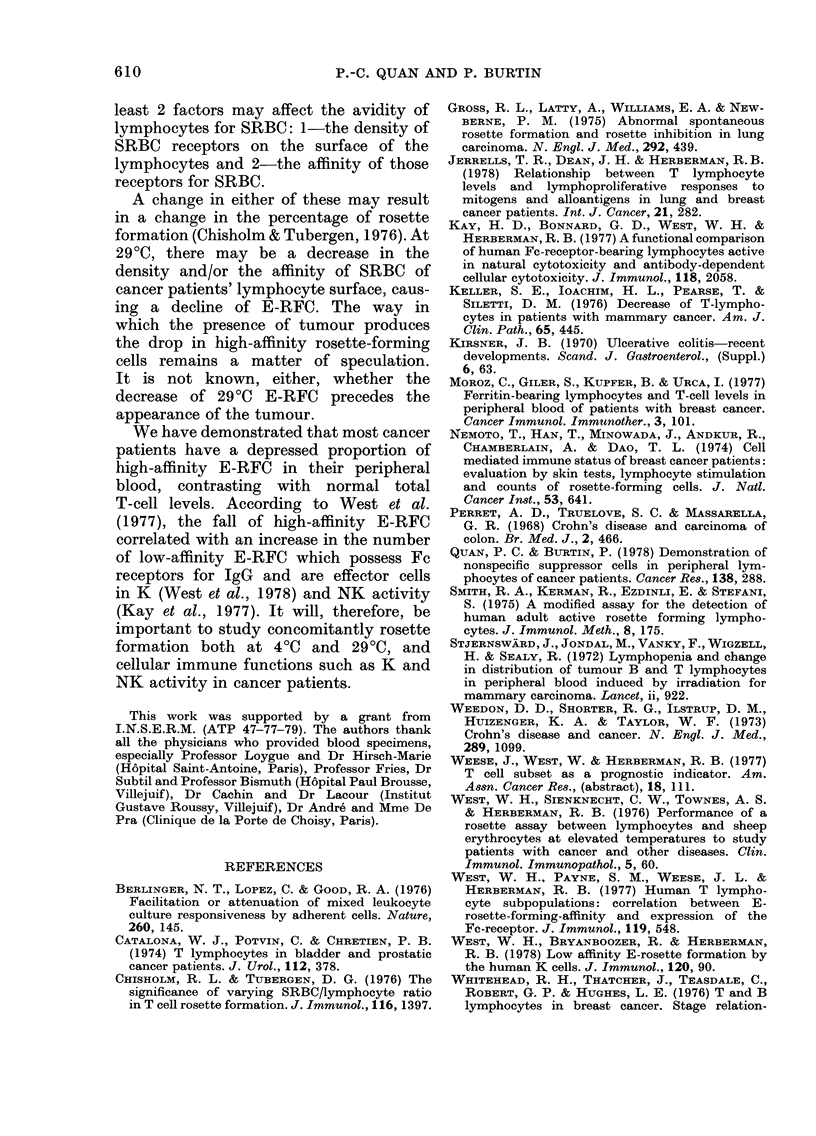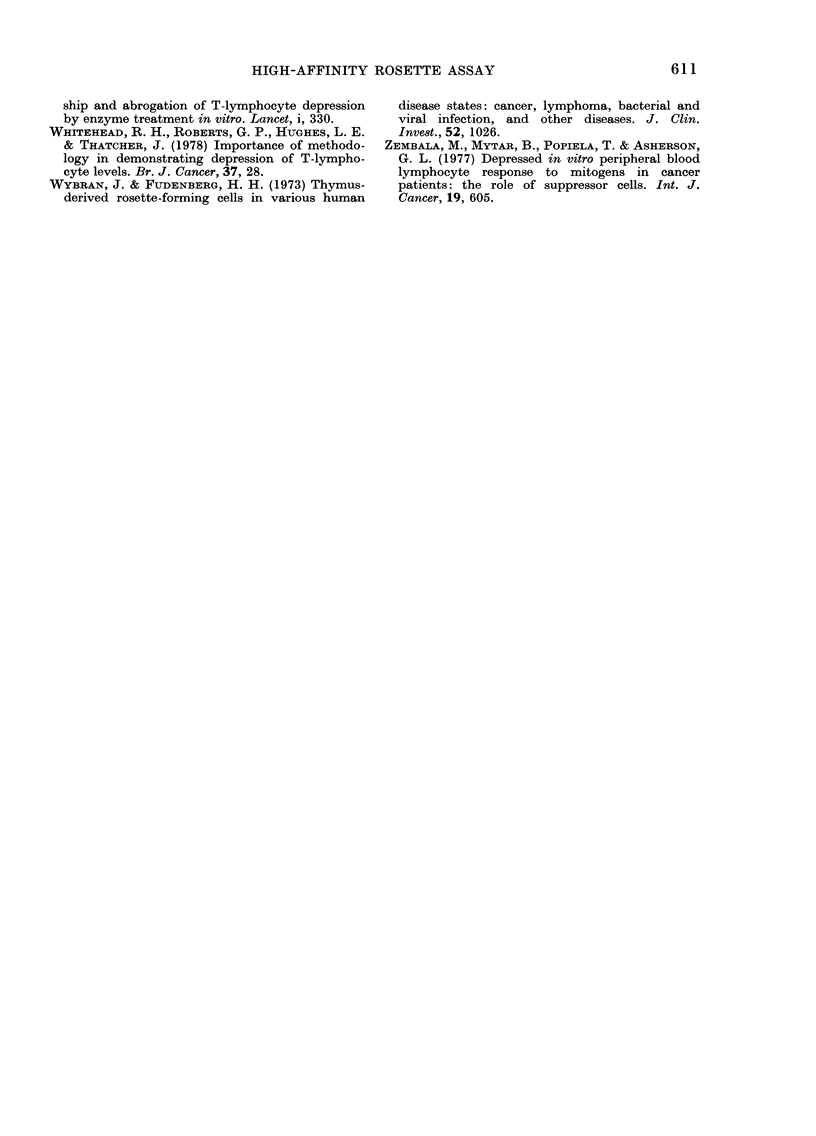# E-rosette-forming cells at 29 degrees C: an assay for the evaluation of the immune status of cancer patients.

**DOI:** 10.1038/bjc.1978.256

**Published:** 1978-11

**Authors:** P. C. Quan, P. Burtin

## Abstract

High-affinity rosette-forming T-cell assays were performed by incubation of peripheral-blood mononuclear cells with sheep erythrocytes (E) at 29 degrees C. As compared with normal controls, the levels of high-affinity rosette-forming cells (RFC) were much more frequently depressed in cancer patients than were the total E-RFC incubated at 4 degrees C. Only 2/83 normal controls had less than 38% 29 degrees C E-RFC (mean 48 +/- 5), whilst 78/89 cancer patients were below this level. The few postoperative patients tested exhibited a normal range of 29 degrees C E-RFC. The 29 degrees C E-rosette assay gives reproducible counts of a T-cell subset, and is a sensitive assay for evaluating the immune status of cancer patients.


*Br. J. Cancer* (1978) **38**, 606

### E-ROSETTE-FORMING CELLS AT 29°C: AN ASSAY FOR THE EVALUATION OF THE IMMUNE STATUS OF CANCER PATIENTS

P.-C. QUAN AND P. BURTIN

*From the Laboratoire d'Immunochimie, Institut de Recherches Scientifiques sur le Cancer, B.P. N°8, 94800 Villejuif, France*

Received 6 July 1978 Accepted 11 August 1978

**Summary.—High-affinity rosette-forming T-cell assays were performed by incubation of peripheral-blood mononuclear cells with sheep erythrocytes (E) at 29°C. As compared with normal controls, the levels of high-affinity rosette-forming cells (RFC) were much more frequently depressed in cancer patients than were the total E-RFC incubated at 4°C. Only 2/83 normal controls had less than 38% 29°C E-RFC (mean 48 ± 5), whilst 78/89 cancer patients were below this level. The few post-operative patients tested exhibited a normal range of 29°C E-RFC.**

**The 29°C E-rosette assay gives reproducible counts of a T-cell subset, and is a sensitive assay for evaluating the immune status of cancer patients.**

HUMAN thymus-derived lymphocytes, or T cells, have been shown to be the principal effectors of cellular immunity. Estimates of circulating T cells, as measured by rosette formation with sheep erythrocytes, (SRBC), suggest that changes in the percentage of rosette-forming cells (E-RFC) may be a reliable index of the level of immuno-competence of the host. However, there are a number of conflicting reports on the proportion of E-RFC in cancer patients. Some authors (Stjernswärd *et al.*, 1972; Nemoto *et al.*, 1974) have reported a normal proportion of T cells in breast-cancer patients, while others described depressed E-RFC in similar patients (Keller *et al.*, 1976; Whitehead *et al.*, 1976; Moroz *et al.*, 1977; Whitehead *et al.*, 1978) and in other malignant diseases (Catalona *et al.*, 1974; Gross *et al.*, 1975).

Other studies have suggested that modification of the rosette technique with suboptimal rosetting conditions apparently permitted better discrimination between normal individuals and cancer patients. Therefore, procedures for detecting a subpopulation of the total E-RFC, termed "active" T-RFC, have been described (Wybran & Fudenberg, 1973; Smith *et al.*, 1975). Wybran & Fudenberg (1973) found that T lymphocytes forming "active" rosettes were diminished in patients with various neoplasms, whereas the total number of T cells was frequently normal in such patients. Recently, West *et al.* (1976) and Weese *et al.* (1977) have found that, with the suboptimal temperature 29°C, a "high-affinity" rosette-forming T-cell assay (29°C E-RFC) was useful in distinguishing between normal individuals and those with cancer and certain other diseases.

The present study was carried out to evaluate the proportion of "high-affinity" E-RFC and 4°C E-RFC in the peripheral blood of cancer patients. We confirmed the findings of West *et al.* (1976) that a decrease in 29°C E-RFC was observed in cancer patients, whereas the level of 4°C E-RFC did not change. 29°C E-RFC assay is much more sensitive, and the level of high-affinity rosette-forming cells reflects better the immune status of cancer patients.

### MATERIALS AND METHODS

*Cancer patients.*—This group consisted of 89 patients, among them 5 in the postoperative period: 37 (22 male, 15 female, 29–88 years old), had histologically proven colorectal cancer, and of these 4 were postoperative; 12 patients (aged 34–68) had breast cancer; the blood samples for assay of these patients were taken at least 3 weeks after chemotherapy and irradiation treatment; and 40 patients (25–65 years old) had various cancers (lung, ovary, stomach, pancreas). One sample was obtained after removal of the tumour.

*Control groups.*—The 83 healthy controls were mainly laboratory personnel and blood donors, 18–60 years old.

For further comparison, 35 hospital patients, 17–73 years old, with various noncancerous diseases (biliary lithiasis, sigmoiditis, chronic ulcerative colitis, Crohn's disease, alcoholic cirrhosis and one case of familial colonic polyposis) were included.

*Lymphocyte separation.*—Ten to 25 ml of heparinized (20 i.u./ml) blood were diluted with Eagle's minimum essential medium (MEM) to 50 ml. The diluted blood was centrifuged on a layer of Ficoll–Triosil (density 1·078) for 15 min at 20°C with 900 *g* at the interface. The layers rich in mononuclear cell were harvested, pooled and centrifuged at 500 *g* for 10 min at 20°C. The lymphocytes were then washed twice with MEM and the final pellet was resuspended at $5 \times 10^6$/ml in RPMI 1640 medium (Eurobio) supplemented with 2 mM glutamine (Grand Island Biological Co., Grand Island, N.Y.), penicillin (100 u/ml) and streptomycin (100 $\mu$g/ml). Cell counting was performed with a haemacytometer using trypan blue in 3% acetic acid solution. This stain provides excellent definition of nuclear and cytoplasmic characteristics. Only those cells with a small round nucleus and relative paucity of cytoplasm were included in the cell counts. The above procedure resulted in a lymphocyte suspension consisting of $> 97\%$ mononuclear cells.

*Preparation of SRBC.*—Sheep blood was obtained fresh each week from the same sheep and stored at 4°C in Alsever's solution (1 :1) and used within 7–10 days. Before use, SRBC were washed 3 times with MEM and adjusted to a 0·5% suspension in RPMI 1640.

*Human AB serum.*—Heat-inactivated AB serum was absorbed against SRBC (v/v) at 20°C for 1 h and 4°C for 30 min before use in the assay.

### *E-rosette techniques*

*Total or 4°C E-RFC.*—Quantified by a minor modification of the method of West *et al.* (1976). Briefly, 0·1 ml of lymphocytes ($5 \times 10^6$/ml) was mixed well with 0·1 ml of AB serum and 0·1 ml of SRBC (0·5%) at a a final SRBC : PBL ratio of 40:1. Tubes were incubated at 37°C for 5 min, centrifuged at 200 *g* for 5 min and the resulting pellet was incubated at 4°C overnight.

*High-affinity or 29°C E-RFC.*—This differed from the total E-RFC assay mainly in that the overnight incubation at 4°C was replaced by incubation at 29°C in a waterbath for 1 h.

E-rosette-forming lymphocytes were quantified in both assays after gentle resuspension of the cell pellets: one from of the suspension was placed on a haemacytometer and the number of rosettes (3 or more SRBC surrounding a lymphocyte) was counted. All tests were performed in duplicate and at least 200 lymphocytes were counted each time.

### RESULTS

### *4°C E-RFC*

As shown in Fig. 1, the mean percentage of 4°C E-RFC in the 83 normal subjects was $65 \pm 6$ (mean $\pm$ s.d.). The lower limit of normal has therefore been defined as the mean $- 2$ s.d., or 53%. Two normal subjects had E-RFC level lower than this. Thirty-five patients with nonmalignant diseases exhibited a mean 4°C E-RFC level of $63 \pm 9$; only one of them, afflicted with alcoholic cirrhosis, had a level lower than 53% (19%). The mean percentage of 4°C E-RFC of the cancer group was $60 \pm 7$; 9/89 had a 4°C E-RFC level below 53%.

### *29°C E-RFC or high-affinity E-RFC*

Figure 2 summarizes all results obtained when assays were performed at 29°C for 1 h. The mean percentage for the normal group was $48 \pm 5$; 4 of these subjects had levels of high-affinity rosettes below the normal range (38%). Furthermore, one normal subject who was followed

FIG. 1.—Distribution of rosette-forming cells at 4°C in normal, benign disease and cancer groups. Means for normal controls ± 2 s.d. are shown by solid and interrupted horizontal lines.

FIG. 2.—Distribution of rosette-forming cells at 29°C in normal, benign-disease and cancer groups. Means for normal controls ± 2 s.d. are shown by solid and interrupted lines.

serially for 5 months presented a minimal variation in 29°C E-RFC values (49, 50, 48, 51, 49). Most of the patients with non-cancerous conditions gave normal values, and the mean level of this group was 43 ± 10. The 7 individuals in this group with low 29°C E-RFC had the following diagnosis: 4 cases of alcoholic cirrhosis at the decompensation stage, one of Crohn's disease (out of 2), one of chronic ulcerative colitis (out of 7), and one of familial colonic polyposis.

There was an abnormal depression of the 29°C E-RFC level in the cancer group (mean 26 ± 10) and most of the cancer patients (78/89) had values below the lower limit of the normal range (38%).

In contrast, the 5 samples obtained in the postoperative period gave values within the normal 29°C E-RFC.

### DISCUSSION

The SRBC rosette constitutes a convenient marker for human T lymphocytes and may reflect their role in immunosurveillance. Thus the proportion of circulating rosette-forming cells may serve as a useful indicator of the immune status of cancer patients. However, there is some disparity in studies of total E-RFC in peripheral blood of cancer patients. Stjernswärd *et al.* (1972) and Nemoto *et al.* (1974) found no significant difference between the levels of E-RFC in breast-cancer patients and normal controls. By contrast, both Keller *et al.* (1976) and Whitehead *et al.* (1976) reported that the percentage was significantly depressed in breast-cancer patients when compared with age-matched controls. Recently Whitehead *et al.* (1978) have demonstrated that the discrepancy in the results for the level of total E-rosette formation in cancer patients is due to incubation time. They found that the levels of E-rosetting cells in both cancer patients and elderly subjects, found low by the usual technique, increased significantly and approached, after overnight incubation at 4°C, the level found in healthy young subjects.

In this study we have found that there is no significant difference with our technique in 4°C E-RFC values between normal subjects and cancer patients. We showed that only 9/89 cancer patients (10%) had a level of 4°C E-RFC below the lower normal limit (53%) defined as the mean value — 2 s.d. The high level of total E-RFC in cancer patients in our studies may be due to the overnight incubation as demonstrated by Whitehead *et al.* (1978); however, with our ratio of SRBC to lymphocytes (40 : 1), there is no effect of incubation time at 4°C on rosette formation, according to Chisholm *et al.* (1976).

It is reported that the depressed response to phytohaemagglutinin and in the MLC of blood lymphocytes in some cancer patients is due to the presence of suppressor cells, even though the total E-RFC is within the normal range (Berlinger *et al.*, 1976; Zembala *et al.*, 1977; Quan & Burtin, 1978). Thus the level of 4°C E-RFC does not always correlate well with the clinical and immune status of patients with malignant diseases. Other studies have suggested that a modification of the rosette technique will allow measurement of a T-cell subpopulation, and that a large proportion of cancer patients can be identified by abnormally low levels of this T subpopulation. Wybran & Fudenberg (1973), using the "active" rosette assay, have demonstrated a decrease in rosette formation by PBL of cancer patients, whereas the majority of these patients had a normal percentage of total E-RFC.

More recently, on the basis of relative affinity for SRBC, West *et al.* (1976) have reported a 29°C rosette assay (with a ratio SPBC/PBL at 120/1) as useful in distinguishing between normal individuals and those with cancer and certain other diseases.

Using the same method, but with a ratio of SRBC/PBL = 40/1, we have demonstrated that patients with malignancies have an abnormally low level of the subpopulation of T cells which forms rosettes at elevated temperatures. Our findings confirmed those of West *et al.* (1976) and Jerells *et al.* (1978). Most cancer patients show a level of high-affinity E-RFC below the lower limit of normal values (38%).

In patients with non-cancerous diseases, there were 7 with a high-affinity RFC level below 38%. Of these 7, 4 were at an advanced stage of alcoholic cirrhosis with markedly abnormal liver function including raised transaminase levels. Of the 3 other patients, one was suffering from chronic ulcerative colitis, one from Crohn's disease and one from familial colonic polyposis. Low levels of 29°C E-RFC in alcoholic-cirrhosis patients have already been reported (West *et al.*, 1976). On the other hand, apart from familial colonic polyposis, which is considered as a precancerous condition, it is noteworthy that chronic ulcerative colitis (Kirsner, 1970) and Crohn's disease (Perret *et al.*, 1968; Weedon *et al.*, 1973) have been reported as increasing the risk of cancer of the large intestine. Yet we do not know that the low level of 29°C E-RFC found in our patients afflicted with one of these diseases is explicable by the presence of an associated carcinoma. Clinical data gave no evidence for such an association.

In the cancer group, we observed some cases with a high level of 29°C E-RFC; 5 of these were postoperative patients. Our findings confirmed those of Weese *et al.* (1977), who have reported that cancer patients with depressed 29°C E-RFC usually showed a return to normal within 3 weeks of surgery. The reproducibility of the technique over time is demonstrated by the serial studies of one normal donor who showed minimal variation in 29°C E-RFC.

What is the mechanism responsible for the depression of high-affinity E-RFC in patients with malignant diseases? West *et al.* (1976) have suggested that an underlying alteration of the T-cell receptor for SRBC in cancer patients and certain other diseases resulted in decreased rosette cohesiveness at raised temperatures. At least 2 factors may affect the avidity of lymphocytes for SRBC: 1—the density of SRBC receptors on the surface of the lymphocytes and 2—the affinity of those receptors for SRBC.

A change in either of these may result in a change in the percentage of rosette formation (Chisholm & Tubergen, 1976). At 29°C, there may be a decrease in the density and/or the affinity of SRBC of cancer patients' lymphocyte surface, causing a decline of E-RFC. The way in which the presence of tumour produces the drop in high-affinity rosette-forming cells remains a matter of speculation. It is not known, either, whether the decrease of 29°C E-RFC precedes the appearance of the tumour.

We have demonstrated that most cancer patients have a depressed proportion of high-affinity E-RFC in their peripheral blood, contrasting with normal total T-cell levels. According to West *et al.* (1977), the fall of high-affinity E-RFC correlated with an increase in the number of low-affinity E-RFC which possess Fc receptors for IgG and are effector cells in K (West *et al.*, 1978) and NK activity (Kay *et al.*, 1977). It will, therefore, be important to study concomitantly rosette formation both at 4°C and 29°C, and cellular immune functions such as K and NK activity in cancer patients.

This work was supported by a grant from I.N.S.E.R.M. (ATP 47–77–79). The authors thank all the physicians who provided blood specimens, especially Professor Loygue and Dr Hirsch-Marie (Hôpital Saint-Antoine, Paris), Professor Fries, Dr Subtil and Professor Bismuth (Hôpital Paul Brousse, Villejuif), Dr Cachin and Dr Lacour (Institut Gustave Roussy, Villejuif), Dr André and Mme De Pra (Clinique de la Porte de Choisy, Paris).

### REFERENCES

Berlinger, N. T., Lopez, C. & Good, R. A. (1976) Facilitation or attenuation of mixed leukocyte culture responsiveness by adherent cells. *Nature*, **260**, 145.

Catalona, W. J., Potvin, C. & Chretien, P. B. (1974) T lymphocytes in bladder and prostatic cancer patients. *J. Urol.*, **112**, 378.

Chisholm, R. L. & Tubergen, D. G. (1976) The significance of varying SRBC/lymphocyte ratio in T cell rosette formation. *J. Immunol.*, **116**, 1397.

Gross, R. L., Latty, A., Williams, E. A. & Newberne, P. M. (1975) Abnormal spontaneous rosette formation and rosette inhibition in lung carcinoma. *N. Engl. J. Med.*, **292**, 439.

Jerrells, T. R., Dean, J. H. & Herberman, R. B. (1978) Relationship between T lymphocyte levels and lymphoproliferative responses to mitogens and alloantigens in lung and breast cancer patients. *Int. J. Cancer*, **21**, 282.

Kay, H. D., Bonnard, G. D., West, W. H. & Herberman, R. B. (1977) A functional comparison of human Fc-receptor-bearing lymphocytes active in natural cytotoxicity and antibody-dependent cellular cytotoxicity. *J. Immunol.*, **118**, 2058.

Keller, S. E., Ioachim, H. L., Pearse, T. & Siletti, D. M. (1976) Decrease of T-lymphocytes in patients with mammary cancer. *Am. J. Clin. Path.*, **65**, 445.

Kirsner, J. B. (1970) Ulcerative colitis—recent developments. *Scand. J. Gastroenterol.*, (Suppl.) **6**, 63.

Moroz, C., Giler, S., Kupfer, B. & Urca, I. (1977) Ferritin-bearing lymphocytes and T-cell levels in peripheral blood of patients with breast cancer. *Cancer Immunol. Immunother.*, **3**, 101.

Nemoto, T., Han, T., Minowada, J., Andkur, R., Chamberlain, A. & Dao, T. L. (1974) Cell mediated immune status of breast cancer patients: evaluation by skin tests, lymphocyte stimulation and counts of rosette-forming cells. *J. Natl. Cancer Inst.*, **53**, 641.

Perret, A. D., Truelove, S. C. & Massarella, G. R. (1968) Crohn's disease and carcinoma of colon. *Br. Med. J.*, **2**, 466.

Quan, P. C. & Burtin, P. (1978) Demonstration of nonspecific suppressor cells in peripheral lymphocytes of cancer patients. *Cancer Res.*, **138**, 288.

Smith, R. A., Kerman, R., Ezdinli, E. & Stefani, S. (1975) A modified assay for the detection of human adult active rosette forming lymphocytes. *J. Immunol. Meth.*, **8**, 175.

Stjernswärd, J., Jondal, M., Vanky, F., Wigzell, H. & Sealy, R. (1972) Lymphopenia and change in distribution of tumour B and T lymphocytes in peripheral blood induced by irradiation for mammary carcinoma. *Lancet*, ii, 922.

Weedon, D. D., Shorter, R. G., Ilstrup, D. M., Huizenger, K. A. & Taylor, W. F. (1973) Crohn's disease and cancer. *N. Engl. J. Med.*, **289**, 1099.

Weese, J., West, W. & Herberman, R. B. (1977) T cell subset as a prognostic indicator. *Am. Assn. Cancer Res.*, (abstract), **18**, 111.

West, W. H., Sienknecht, C. W., Townes, A. S. & Herberman, R. B. (1976) Performance of a rosette assay between lymphocytes and sheep erythrocytes at elevated temperatures to study patients with cancer and other diseases. *Clin. Immunol. Immunopathol.*, **5**, 60.

West, W. H., Payne, S. M., Weese, J. L. & Herberman, R. B. (1977) Human T lymphocyte subpopulations: correlation between E-rosette-forming-affinity and expression of the Fc-receptor. *J. Immunol.*, **119**, 548.

West, W. H., Bryanboozer, R. & Herberman, R. B. (1978) Low affinity E-rosette formation by the human K cells. *J. Immunol.*, **120**, 90.

Whitehead, R. H., Thatcher, J., Teasdale, C., Robert, G. P. & Hughes, L. E. (1976) T and B lymphocytes in breast cancer. Stage relationship and abrogation of T-lymphocyte depression by enzyme treatment *in vitro*. *Lancet*, i, 330.

WHITEHEAD, R. H., ROBERTS, G. P., HUGHES, L. E. & THATCHER, J. (1978) Importance of methodology in demonstrating depression of T-lymphocyte levels. *Br. J. Cancer*, **37**, 28.

WYBRAN, J. & FUDENBERG, H. H. (1973) Thymus-derived rosette-forming cells in various human disease states: cancer, lymphoma, bacterial and viral infection, and other diseases. *J. Clin. Invest.*, **52**, 1026.

ZEMBALA, M., MYTAR, B., POPIELA, T. & ASHERSON, G. L. (1977) Depressed *in vitro* peripheral blood lymphocyte response to mitogens in cancer patients: the role of suppressor cells. *Int. J. Cancer*, **19**, 605.